# The interplay among conflict, water scarcity, and cholera in Northern Nigeria

**DOI:** 10.1002/puh2.118

**Published:** 2023-08-19

**Authors:** Bashar Haruna Gulumbe, Zaharadeen Muhammad Yusuf, Abdullahi Adamu Faggo, Tajudeen O. Yahaya, Sahabi Sule Manga

**Affiliations:** ^1^ Department of Microbiology Faculty of Science Federal University Birnin‐Kebbi Birnin Kebbi Kebbi Nigeria; ^2^ Department of Biochemistry Faculty of Science Alqalam University Katsina Katsina Katsina Nigeria; ^3^ Department of Microbiology Bauchi State University Gadau Bauchi State Nigeria; ^4^ Department of Biological Sciences Faculty of Science Federal University Birnin‐Kebbi Birnin Kebbi Kebbi Nigeria; ^5^ Department of Microbiology Kebbi State University of Science and Technology Aliero Kebbi Nigeria

**Keywords:** armed conflict, Boko Haram, cholera, displacement, humanitarian crises, Nigeria, water scarcity, waterborne diseases

## Abstract

Cholera is a recurring issue in Nigeria, with outbreaks predominantly affecting the Northern states. In 2021, Nigeria experienced its most significant cholera epidemic in a decade, resulting in thousands of fatalities and cases. The spread of cholera and other waterborne diseases in Nigeria is linked to various factors such as flooding, conflict, and water scarcity. The country's poor drinking water quality and lack of equitable access to clean water exacerbate the problem, particularly in the conflict‐affected areas of the North. This paper discusses the interaction of conflict‐related displacement, water shortages, and cholera transmission in Northern Nigeria and provides insights into how these factors impact water resources and public health. This information can inform initiatives and policies to reduce the impact of conflict on water resources and enhance access to clean water in impacted areas. The article suggests that the primary challenges in eliminating cholera in the region include a lack of information due to severe security situations, bureaucratic requirements for delivering essential supplies, and a chronic shortage of water. A successful response to cholera in conflict and displacement situations also requires community engagement and the security and safety of humanitarian personnel. Long‐term solutions to the uprisings in the region are necessary to put an end to the issues related to banditry and insurgency.

## INTRODUCTION

**FIGURE 1:**
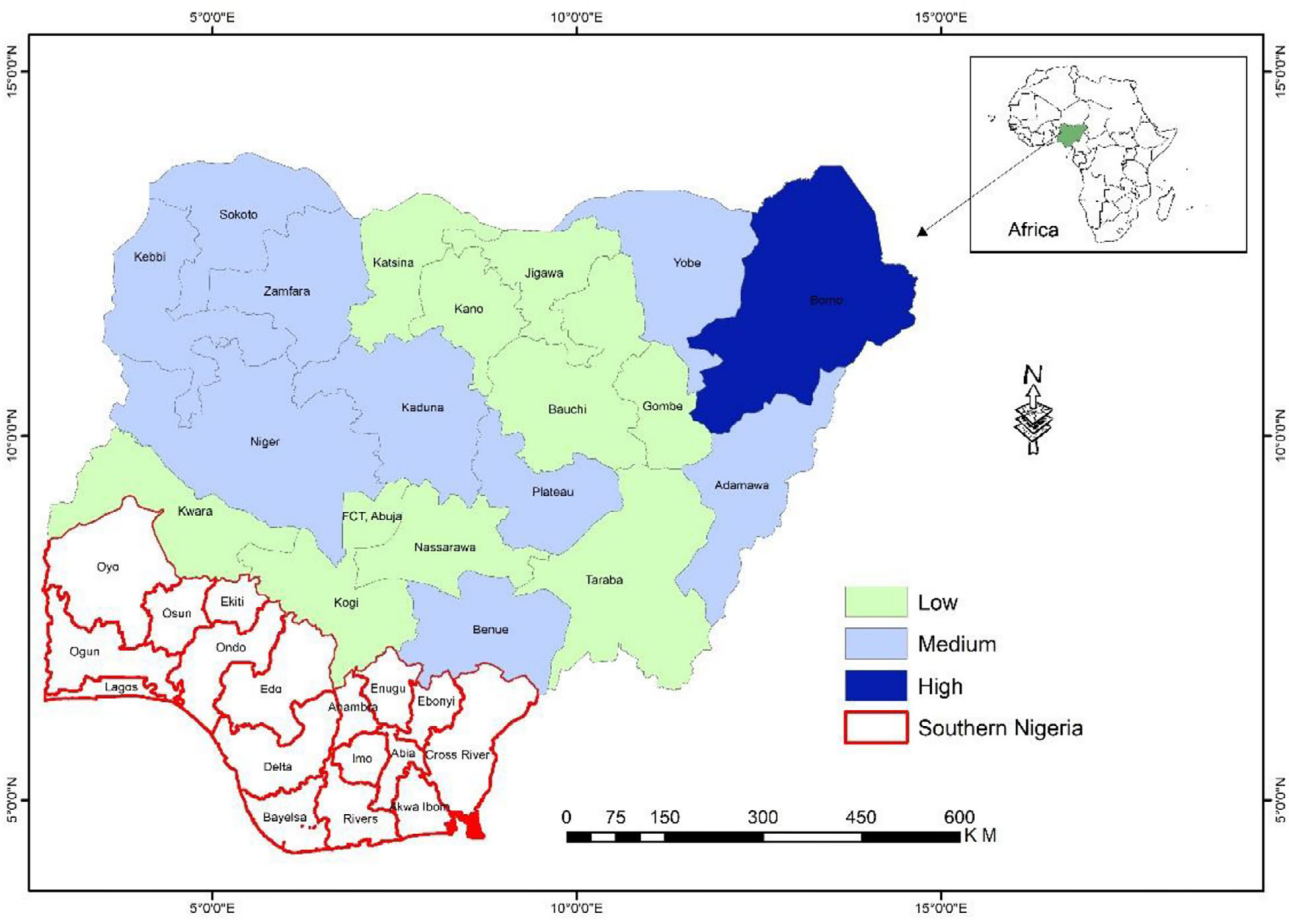
Map of Nigeria showing Northern states affected by armed conflicts, February 2023.

Since its documented emergence in 1972, cholera has sporadically affected Nigeria, with outbreaks particularly prevalent in the Northern states. The country faced its biggest cholera epidemic in a decade in 2021 [1]. The Nigeria Centre for Disease Control (NCDC) recorded 3604 fatalities and a total of 109,189 suspected cases as of December 19, 2021 [2]. As of November 2022, a total of 23,550 cases were documented by the NCDC with the Northern states of Borno (12,459), Yobe (1888), Katsina (1632), Gombe (1407), Taraba (1142), and Kano (1131) accounting for 84% of all cumulative cases [3]. The spread of cholera, alongside other waterborne diseases, has been associated with multiple factors [4]. These include flooding, conflict‐induced displacement, and scarcity of clean drinking water, which is essential for hygiene and sanitation [5, 6]. In Nigeria, achieving equitable and sustainable access to clean drinking water remains challenging due to the country's poor drinking water quality and lack of equity in access. Almost 86% of Nigerians lack access to a source of safely managed drinking water, even though it is estimated that roughly 70% of the population has access to basic water services. More than half of these water sources are polluted [7]. This problem is compounded by conflicts and displacement. Large populations are now affected by persistent conflict and displacement in Northern Nigeria, perpetuated by Boko Haram in the Northeast to banditry in the Northwest and Central (Figure 1), bringing about a catastrophic humanitarian crisis.

Access to water supplies is significantly impacted by attacks on infrastructure and population displacement in these settings, which in turn worsens the spread of cholera and other waterborne illnesses. Consequently, a study on how conflict‐related displacement, water shortages, and cholera transmission interact in Northern Nigeria can add to the body of knowledge on how these factors affect water resources and public health. This information may be applied to initiatives and policies aiming to reduce conflict's effect on water resources and enhance access to clean water in impacted areas.

## CONFLICT, WATER CRISIS, AND CHOLERA SURGE

**FIGURE 2:**
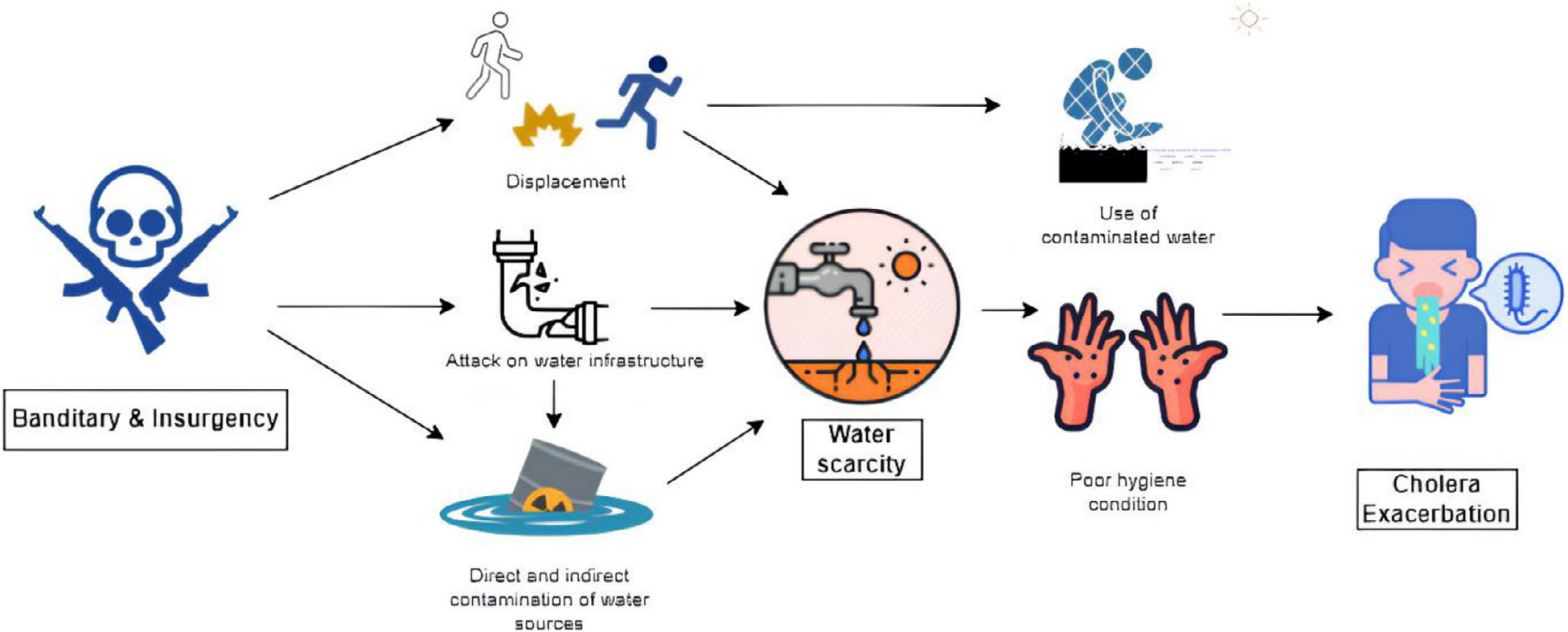
A schematic representation of how conflicts and water scarcity have led to a surge in cholera in Nigeria.

The complex interplay between water scarcity and conflict‐induced displacement in Northern Nigeria significantly exacerbates cholera outbreaks. Numerous interconnected factors contribute to this water scarcity. These include the destruction of water infrastructure, pollution of water sources, forced migration leading to increased demand for water, disruption of water distribution networks, poor water management, limited access to water, destruction of irrigation systems, diversion of water resources, and disruption of international water agreements [8]. These factors often deplete water supplies and impede their effective distribution, aggravating water shortages in the affected areas (Figure 2). In certain cases, resource scarcity, such as water, can incite violent conflicts [9, 10], resulting in displacement, contamination of resources, and subsequent outbreaks of infectious diseases. An apt example is the situation in the Lake Chad Basin, where dwindling water resources have escalated competition and incited clashes among local communities and cross‐border tensions [11]. Conflict‐induced displacement and water scarcity create a conducive environment for cholera transmission [12]. Displaced individuals often live in overcrowded, unhygienic conditions with limited access to clean water and sanitation [6].

Ongoing conflicts, including the 14‐year conflict with Boko Haram and the increasing violence from highly sophisticated criminal gangs in the Northwest and Northcentral regions, have devastated communities across Northern Nigeria [13]. These conflicts have caused significant public infrastructure damage and displaced millions of people [13, 14, 15, 16]. Such forced relocations often lead to regions with scarce water resources, exacerbating the competition for water and its scarcity. Attacks have destroyed water infrastructure, such as wells and boreholes, and power supplies, depriving many residents of the affected regions of access to clean drinking water. Fear, spurred by frequent kidnappings associated with these conflicts, inhibits residents, including those in charge of municipal water resource management, from venturing out to fetch water, further limiting access to clean water [17]. From 2011 to 2019, in Zamfara State alone, up to 3600 individuals were kidnapped, and 8000 people were killed [18]. In addition, movement restrictions imposed by authorities in response to these conflicts potentially hinder people from leaving their homes to source water [6]. These interlinks undoubtedly influence cholera outbreaks in the region, contributing to a dramatic increase in the number of recorded cases in recent years [13, 14, 15, 16].

## CHOLERA TRANSMISSION IN NORTHERN NIGERIA

Cholera, due to its high prevalence and significant fatality rates, constitutes a major public health concern. Over the last several decades, Nigeria has experienced numerous cholera outbreaks. In 2010, over 40,000 cases were reported, resulting in 1700 fatalities, whereas in 2018, the case count rose to over 50,000, with 800 fatalities. The situation further intensified between October 2020 and October 2021, when approximately 93,000 cholera cases and 3000 fatalities were recorded [19]. Throughout these outbreaks, Northern Nigeria, particularly the Northeast—severely affected by the insurgency—reported the majority of the cases. As of December 19, 2021, the overall reported statistics stood at 3604 fatalities and 109,189 suspected cases. Furthermore, by November 2022, the NCDC reported an additional 23,550 cases. Of these, six Northern states accounted for 84%. These include Borno (12,459 cases), Yobe (1888 cases), Katsina (1632 cases), Gombe (1407 cases), Taraba (1142 cases), and Kano (1311 cases) [3]. This alarming trend underscores the critical need for targeted interventions to control and prevent cholera outbreaks [3].

This consistent trend in cases in the region explains the fact that in addition to other factors such as poor sanitation, limited access to healthcare, and flooding [12], conflict and water scarcity have a compounding impact on cholera epidemiology in the region. Some of the places hit hard by conflicts are challenging to reach and have poor water sanitation and hygiene infrastructure and medical services as a result of the instability [13, 15, 20]. The issue is made worse by the displacement of internally displaced persons (IDPs) and the lack of proper sanitation facilities in congested communities, which led to latrine pits overflowing. This situation provides a viable breeding ground for infectious disease transmission including cholera.

## TACKLING CHOLERA IN THE REGION

The government collaborates closely with nongovernmental organizations (NGOs) to fight cholera by building reference labs, managing disease monitoring, and providing other services [21]. Nigeria launched the “National Strategic Plan of Action on Cholera Control” to identify and provide vaccines in cholera‐impacted regions by World Health Organization (WHO) recommendations to identify hotspot areas wracked by the disease [22]. In Bauchi State alone, these interventions have facilitated the effective immunization of approximately 710,212 individuals [23].

To bolster the fight against cholera, the National Cholera Emergency Operations Center (EOC) was revitalized in 2021, which resulted in huge assistance from the quick response teams in the hotspot regions of Kaduna, Kano, Bauchi, Plateau, and Benue. The EOC enhanced the planning, administration, and distribution of oral cholera vaccines in IDP camps, and they regularly track cholera incidence [22].

The government's efforts also extend to ensuring access to clean drinking water, promoting good sanitation, and instilling personal hygiene practices to curb the spread of cholera [2]. For instance, to combat the cholera epidemic in Borno State in 2019, Nigeria collaborated with the International Organization for Migration to provide more than 68 boreholes [24]. NGOs also give the government crucial data on the prevalence of several infectious illnesses in Nigeria as well as the provision of vaccination support [14]. For instance, Gavi, the Vaccine Alliance, United Nations International Children's Emergency Fund (UNICEF), and WHO support efforts to control cholera in Northern Nigeria [2]. In addition to providing logistical support in the battle against cholera for various states in the Northwest and Northeast, UNICEF also funds television and radio campaigns to raise awareness of the disease [6]. Despite these efforts, however, cholera outbreaks continue to persist with an increase in prevalence, which can be attributed to several factors, including poor sanitation, limited access to healthcare, flooding, and conflict‐related displacement [6].

## ADDRESSING CHOLERA DURING CONFLICT

During violence and migration, cholera control presents a variety of intricate problems. Insecure living conditions, inadequate sanitation, overcrowding, interrupted health services, a lack of resources, and restricted access to clean water all pose serious obstacles to cholera prevention and control in conflict‐affected communities [12, 14, 15]. The issue is made worse by the breakdown of fundamental infrastructure and disruptions in the water delivery networks.

Moreover, safety concerns for healthcare workers and NGOs active in these areas pose a substantial barrier to effective cholera control. Fear, often prevalent in conflict‐ridden communities, may prevent individuals from seeking necessary care, thereby contributing to the propagation of the disease [6]. Worsening poverty and preexisting vulnerabilities, often aggravated by a lack of employment opportunities and income generation, further compound these issues [6]. The intersectionality of these factors creates a complex scenario for cholera control during times of conflict, requiring integrated and multifaceted intervention strategies.

## CONCLUSION

One of Nigeria's key public health issues is cholera, especially in the country's troubled North. Cholera epidemics, armed conflict, and water scarcity are all closely related in this area. The primary obstacles to the elimination of cholera in the region still include a lack of information owing to severe security situations and restrictions on transportation, displacement, and water scarcity. To effectively resolve issues related to banditry and insurgency, it is imperative to have a comprehensive strategy in place, which includes long‐term solutions to these uprisings. Consequently, this requires strengthening infrastructure for healthcare, humanitarian aid, as well as water and sanitation services to tackle these challenges head‐on. To sustain and improve present responses, the budgetary deficiencies in the health sector must also be rapidly addressed. A successful response to cholera in war and displacement situations also requires community participation and engagement, as well as the security and safety of humanitarian personnel.

## AUTHOR CONTRIBUTIONS

*Conceptualization; writing; reviewing and figures design*: Bashar Haruna Gulumbe. *Writing; reviewing and approval of the manuscript*: all authors.

## CONFLICT OF INTEREST STATEMENT

The authors declare that there is no conflict of interest that could be perceived as prejudicing the impartiality of the research reported.
